# Cholesterol-lowering effects and potential mechanisms of chitooligosaccharide capsules in hyperlipidemic rats

**DOI:** 10.29219/fnr.v62.1446

**Published:** 2018-06-14

**Authors:** Yao Jiang, Chuhan Fu, Guihua Liu, Jiao Guo, Zhengquan Su

**Affiliations:** 1 Guangdong Engineering Research Center of Natural Products and New Drugs, Guangdong Pharmaceutical University, Guangzhou, China; 2 Guangdong Metabolic Diseases Research Center of Integrated Chinese and Western Medicine, Key Unit of Modulating Liver to Treat Hyperlipemia SATCM (State Administration of Traditional Chinese Medicine), Guangdong Pharmaceutical University, Guangzhou, China; 3 Shenzhen Center for Disease Control and Prevention, Shenzhen, China

**Keywords:** chitosan oligosaccharide, antihyperlipidemic, gene difference expression, high-fat diet, CYP7A1, HMGCR

## Abstract

**Background:**

Chitooligosaccharide (COS) has shown potential antihyperlipidemic activity in a few studies as a functional food.

**Method:**

We investigated the cholesterol-lowering effect and potential mechanisms of chitooligosaccharide capsules (COSTC) in male SD rats fed a high-fat diet.

**Results:**

COSTC could ameliorate serum lipid levels. Simultaneously, the cholesterol-lowering effect is probably attributed to its role in two pathways: upregulating the gene expression and activity of cholesterol 7α-hydroxylase (CYP7A1), liver X receptor alpha (LXRA), and peroxisome proliferation activated receptor-α (PPARα), which facilitates the conversion of cholesterol into bile acid; downregulating the gene expression and activity of enzymes including 3-hydroxy-3-methylglutaryl-coenzyme A reductase (HMGCR) and sterol-responsive element binding protein-2 (SREBP2) and upregulating the low-density lipoprotein receptor (LDLR) to reduce the denovo synthesis of cholesterol.

**Conclusion:**

Studies have suggested that COSTC has potential usefulness as a natural supplement or functional food for preventing and treating hyperlipidemia.

With the rapid development of the economy, many people have experienced improvements in living standards, and thus, the proportion of high-fat diet increases with each passing day. The long-term intake of high-fat food disrupts internal lipid metabolism, appearing as a decrease in high-density lipoprotein cholesterol (HDL-C) and an increase in total cholesterol (TC), triglyceride (TG), and low-density lipoprotein cholesterol (LDL-C), eventually resulting in fat accumulation, obesity, and hyperlipidemia (1). Hyperlipidemia is an important risk factor for the development and progression of atherosclerosis and subsequent cardiovascular disease, which is one of the most serious diseases of mankind (2).

In recent decades, seek for new drugs for ameliorating serum lipid profiles (reducing TG and TC levels) and/or targeting lipid metabolic-related factors (3–8), such as CYP7A1, HMGCR, proprotein convertase subtilisin/kexin type 9 (PCSK9), and LDLR, has received increasing attention. Although lipid-lowering medicines are primarily chemical drugs with good effects on the market, the adverse effects of these chemicals are problematic (9, 10). Given the side effects of the current antihyperlipidemic drugs, the development of natural products as alternative sources of antihyperlipidemic functional foods or agents remains an urgent necessity. Accumulating studies have indicated the significant cholesterol-lowering activities of natural agents, particularly polysaccharides, which have been used in preventing obesity and improving plasma lipids (11–13).

COS is an oligosaccharide easily obtained through acid hydrolysis or enzymatic hydrolysis from chitosan (14, 15). The family of COS compounds which contains between 2 and 10 glucosamine residues that are attached through β-d-(1–4) glycoside linkages have received much attention because of their small molecular weight, good aqueous solubility, and diverse biological activity (16–19). Previous studies have shown that chitosan oligosaccharide with a molecular weight ≤1000 Da (COST, Fig. 1) shows improvements in lipids and total bile acids (TBA) levels in hyperlipidemic rats (20). Previous studies have also shown that COST has effects on the inhibition of 3T3-L1 preadipocyte differentiation and enhancement of hepatic function (21–24). However, until recently, the in-depth mechanisms of cholesterol-lowering and hepatoprotective activities of COST remain unclear. Digital gene expression (DGE) tag profiling is a powerful method for evaluating differences in gene expression with high repeatability in a chosen cell, tissue, organism, or condition. Gene ontology is an internationally accepted gene function analysis that represents the molecular function of the gene, including the biological processes involved and cell location (25).

**Fig. 1:**
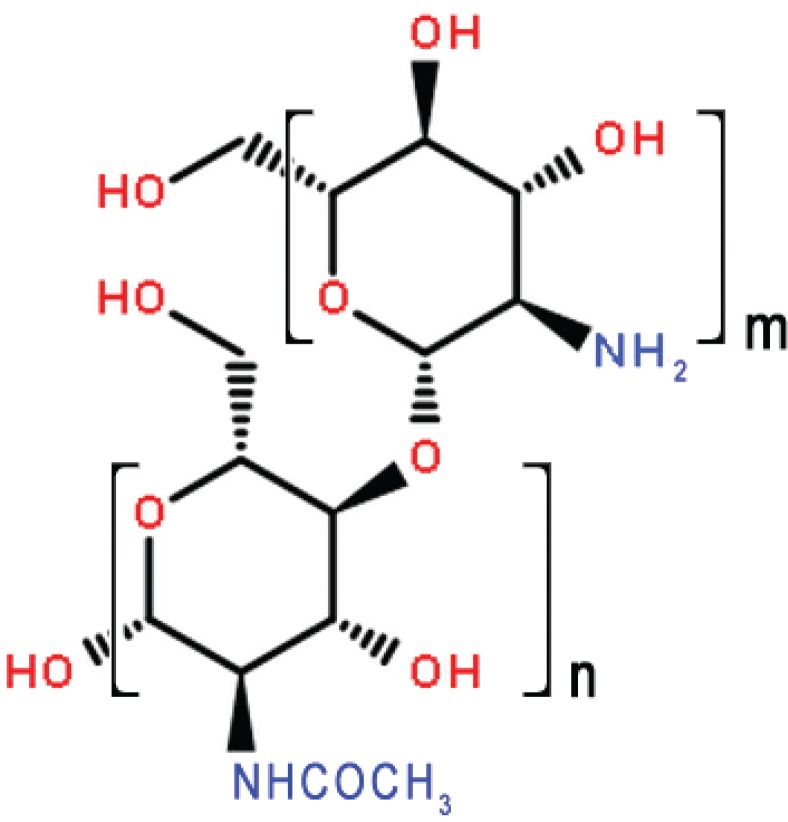
The molecular structures of COST (*n* = 2–10).

In this study, we investigated COST capsules (COSTC), prepared according to a previous study (26), which have several advantages: (1) enhancing the stability of COST, which shows strong hygroscopicity and oxidability; (2) boosting the action rate of COST, because the capsule is rapidly dissolved, dispersed, and assimilated in the gastrointestinal tract; and (3) these molecules are easy to identify and administer to most people. Based on the analysis of differential gene expression in the liver tissues of hyperlipidemic rats, which was closely related to the hepatic hyperlipidemic response, we investigated the antihyperlipidemic effects of COSTC using high-fat diet-induced rats, and the results demonstrated that COSTC can ameliorate lipid metabolism and its related complications. However, fewer studies have been reported on the aspect of the antihyperlipidemic mechanism of COSTC in the liver. Therefore, in this study, we demonstrated that COSTC improved lipid metabolism via the upregulation of the gene expression and activity of CYP7A1, LXRA, and PPARα, which promotes the conversion of cholesterol into bile acid, downregulating the gene expression and activity of enzymes, including HMGCR and SREBP2, and upregulating LDLR to reduce the *de novo* synthesis of cholesterol. Thus, these results show that COSTC may be a prospective functional food and agent for antihyperlipidemic prevention or treatment.

## Materials and Methods

### Materials

Commercial samples of COST (Mw ≤ 1,000 Da; degree of deacetylation, 95.6%; lot: 160926C) used in this study were obtained from Aokang (Shangdong, China). Atorvastatin calcium tablets were supplied from Pfizer Pharmaceutical Company Limited (Daolian, Liaoning, China). Povidone and carboxymethylcellulose sodium were obtained from Tianjin Kermel Chemical Reagent Co., Ltd. (Tianjin, China). TC, TG, HDL-C, LDL-C, aspartate aminotransferase (AST), and alanine aminotransferase (ALT) kits were purchased from BioSino Biotechnology and Science, Inc. (Beijing, China). The COSTC used in this study were prepared in the laboratory.

### Animals and Diets

Eighty healthy male Specefic Pathogen Free (SPF) Sprague-, Dawley rats (weight, 200 ± 20 g; age, 8 weeks; No.44007200034526) were used for the animal experiments and were obtained from the Guangdong Medical Laboratory Animal Center (GMLAC, Guangzhou, China). The animals were fed in an SPF room (temperature: 22–25°C; related humidity: 50–70%; differential pressure: ≥ 10 Pa; 12:12 h light/dark cycle). All animal experimental protocols were approved through the Institutional Animal Care and Use Committee of Guangdong Pharmaceutical University (Guangzhou, China). Water was *arbitrarily* provided throughout the experiments. All animals were fed a normal diet (Guangdong Pharmaceutical University Laboratory Animal Center, Guangzhou, China) for 1 week of adaptive feeding. Subsequently, 10 rats continued to be fed the normal diet as a control group (NF), and the remaining rats were fed high-fat diets to achieve the hyperlipidemia model.

The basic diet was composed of crude protein (20.0%), crude fiber (4.8%), total crude fat (4.3%), moisture (9.7%), calcium (1.19%), phosphorus (0.77%), Ca^2+^/P^5-^ = 1.55, and crude ash (6.6%). The test results showed that total bacterial count (B10 cfu/g), total molds and yeasts (B10 cfu/g), the *Escherichia coli* count (B3.0 MPN/100 g), and *Salmonella* (not detected) all met the stipulated standards. The high-fat diet included basic feed (52.6%), sucrose (20.0%), lard (15.0%), cholesterol (1.2%), bile salts (0.2%), casein (10%), calcium hydrophosphate (0.6%), and mountain flour (0.4%) in SPF packaging (provided by GMLAC, No. 20160939). After 2 weeks, blood samples were collected from the orbital vein using a capillary under ether anesthesia. The blood samples were centrifuged (4°C; 3,500 r × min^-1^; 15 min) to obtain plasma samples to determine serum TG, TC, and LDL-C levels, and in the model group versus control group, the differences in the TC, TG, or LDL-C levels were significant, thereby establishing a judgment model.

Fifty rats were divided into 5 stochastically selected groups (*n* = 10 rats per group): (1) high-fat diet feeding group (HF); (2) high-fat diet feeding administered with atorvastatin (7 mg/kg·day) (AVT); (3) high-fat diet feeding administered with a low dose of COSTC (150 mg/kg·day) (COSTC-L); (4) high-fat diet feeding administered with an intermediate dose of COSTC (300 mg/kg·day) (COSTC-M); and (5) high-fat diet feeding administered with a high dose of COSTC (600 mg/kg·day) (COSTC-H) according to a previous study (27). The test samples, dissolved in water, were treated orally through gavage with a simultaneous dose of 1 mL/100 g per day for 6 weeks. The rats in the NF and HF groups were administered an equal capacity of solvent.

## Experimental Design

### Determination of Food Intake, Weight Gain and Parameters of Viscera

A record of the food intake levels for all rats was maintained daily, and the body weight was measured weekly during the experimental period. All rats obtained food and water *ad libitum*. At the end of experimental period, the rats were fasted for 24 h and subsequently subjected to 1% sodium pentobarbital (0.5 mL/100 g BW) anesthesia, and the blood samples were assembled from the abdominal aorta. Then, the heart, liver, kidneys, epididymal white adipose tissue, and perirenal white adipose tissue of rats were quickly harvested and the samples weighed on electronic scales. The total wet weight of the epididymal and perirenal fat samples was used to measure the body fat ratio. The wet weight of the liver was analyzed to determine the visceral index. The tissues were promptly stored at -80°C until further analysis.

### Serum Biochemical Analysis

Blood samples were collected by cardiac puncture and subsequently centrifuged; the serum was stored at 20°C. The concentrations of TG, TC, LDL-C, and HDL-C in the serum were analyzed using commercial assay kits and an automated biochemistry analyzer BC200 instrument (BC200, Beijing Precil Instrument Co. Ltd., Beijing, China) according to the manufacturer’s instructions. The activities of plasma ALT and AST were analyzed using ALT and AST assay kits, respectively, and a Mithras LB 940 Multimode Microplate Reader (Berthold Technologies GmBH & Co. KG, Germany).

### Histological Staining

The liver, kidney, white epididymal, perirenal, and subcutaneous adipose tissues samples (about 0.5 cm^3^) were taken, washed with pre-cooling saline, placed in the embedding box, labeled with a pencil on the embedding box, and then placed in 12% formaldehyde solution. After immobilization, the ethanol was gradually dehydrated at a concentration of 30–100%. After impregnation, the paraffin was embedded and then sliced on a slicer, and subsequently cut into 3–5-μm-thick sections using a microtome (Leica RM2235; Leica, Heidelberg, Germany). The tissues were stained with hematoxylin and eosin (H&E) and observed under a microscope at 200× magnification.

### Digital Gene Expression Tag Profiling

**Fig. 2:**
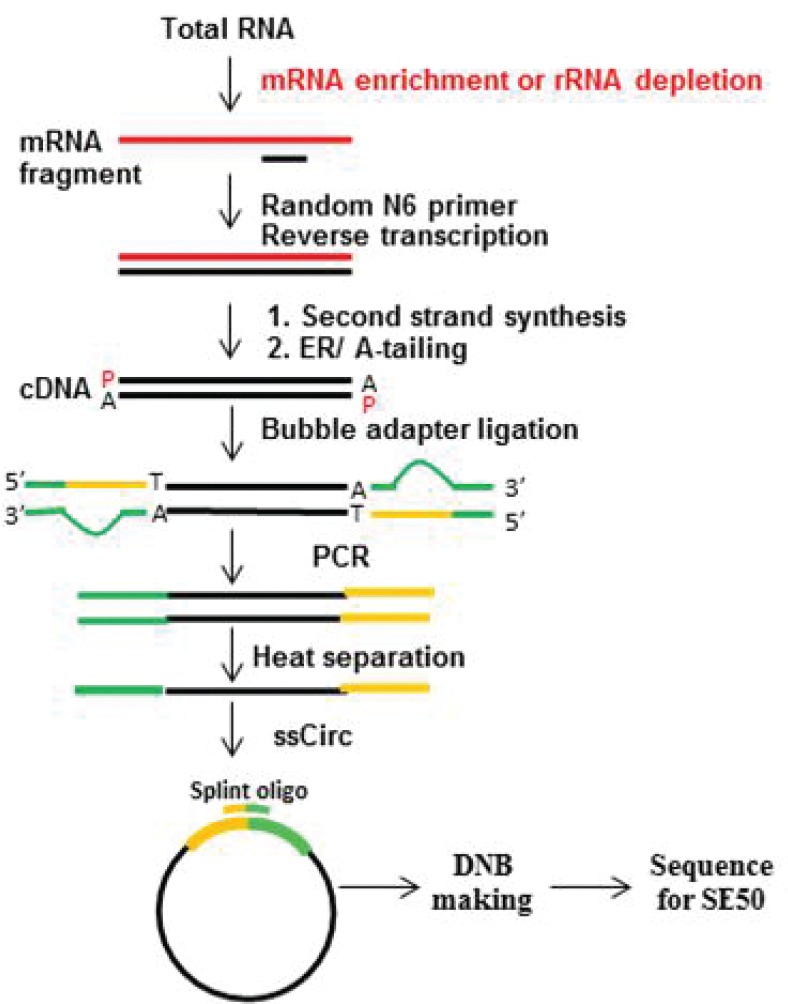
RNA-Seq experimental process. Hybridize the rRNA with the DNA probe and subsequently digest the DNA/RNA hybrid strand, followed by DNase I digestion to remove the DNA probe. Obtain the target RNA through purification. Fragment the target RNA and perform reverse transcription to double-stranded cDNA (dscDNA) using the N6 random primer. End repair the dscDNA with phosphate at the 5'-end, and add a sticky ‘A’ tail at the 3'-end. Subsequently, ligate the adaptor at the 3'-end of the dscDNA using a sticky ‘T’ tail. Amplify the ligation product using two specific primers. Denature the PCR product by heating, and cyclize the single-stranded DNA using a splint oligo and DNA ligase.

The liver RNA in HF and COSTC groups was extracted using Agilent RNA 6000 Pico kits (Agilent Technologies, Santa Clara, CA, USA). The concentration and quality were detected using an Agilent 2100 instrument (Agilent Technologies, Santa Clara, CA, USA). The Beijing Genomics Institution conducted the DGE. The experiment pipeline is described in Fig. 2. Briefly, the rRNA was hybridized with DNA probe, and the DNA/RNA hybrid strand was digested, followed by a DNase I reaction to remove DNA probe. Subsequently, the target RNA was obtained after purification. The target RNA was fragmented and reverse transcribed to double-stranded cDNA (dscDNA) using an N6 random primer. The PCR product was denatured using heat, and the single-stranded DNA was cyclized using a splint oligo and DNA ligase. The prepared library was sequenced, and subsequently, the differentially expressed genes were screened using the NOISeq method and clustering analysis of differentially expressed genes, Gene Ontology.

### Quantitative RT-PCR

Total RNA was isolated from the liver tissues of rats using TRIzol reagent (Invitrogen, Inc., USA). Single-stranded cDNA was generated using the PrimeScript^TM^ RT Reagent kit with gDNA Eraser (TaKaRa, Lot: D413KA5332, Shiga, Otsu, Japan) and stored at -20°C. The cDNA products were amplified using real-time RT-PCR and the TaKaRa SYBR Premix Ex Taq™ kit (TaKaRa, Lot: AK7103, Shiga, Otsu, Japan) as well as the Bio-Rad IQ5 real-time PCR system and analysis software (Applied Biosystems, Carlsbad, CA, USA). After initial denaturation at 95°C for 30 s, PCR amplification was performed using 40 cycles at 95°C for 3 s, 60°C for 20 s, and 65°C for 15 s. The nucleotide sequences used for PCR were designed and synthesized at Sangon Biotech Co. Ltd. (Shanghai, China). Beta-actin (β-actin) was used as the internal control (housekeeping gene). The relative quantification of mRNA expression was analyzed using the delta CT method. The following primer pairs were used for PCR: 5'-ACCTGCCGGTACTAGACAGC-3' and 3'-CAGGACATATTGTCGCGCCT-5' for CYP7A1; 5'-CTGCAACGGAGTTGTGGAAG-3' and 3'-TCGCAGCTCAGCACATTGTA-5' for LXRA; 5’-GCCGACCTGACGAATTCCAG-3’ and 3'-ATCCGACCAGTCACGACAGT-5' for LDLR; 5'-GGAGACCATGGAGACCCTCAC-3' and 3'-AGACAATGGGACCTGGCTGAA-5' for SREBP2; 5'-CCTCCATTGAGATCCGGAGGA-3' and 3'-ACAAAGAGGCCATGCATACGG-5' for HMGCR; and 5'-TCTGAACATTGGCGTTCGCA-3' and 3'-TCCCTCAAGGGGACAACCAG-5' for PPARα.

### Western Blotting

Total proteins were isolated from liver tissues (50 mg) using 1.0 mL of cold RIPA lysis buffer (50 mM Tris, pH 7.4, 150 mM NaCl, 1% Triton X-100, 1% sodium deoxycholate, 0.1% SDS, and protease and phosphatase inhibitor), followed by centrifugation twice at 12,000×g for 20 min at 4°C, and the protein concentration was measured using the BCA Protein Assay Kit (Beyotime, Lot: 0907A16, Shanghai, China). The isolated proteins were diluted to the same protein concentrations. The extracted proteins were diluted to the same protein concentrations. Equal amounts of each protein lysate were electrophoresed via sodium dodecyl sulfate-polyacrylamide gel electrophoresis (SDS-PAGE) and electrophoretically transferred onto polyvinylidene difluoride (PVDF) membranes (Millipore Corp., Billerica, MA, USA). After sealing with 5% bovine serum albumin (BSA) or skim milk powder in TBST buffer (25 mM Tris, 150 mM NaCl, and 0.05% Tween 20, pH 7.2–7.5), the membranes were hatched with primary antibodies, including HMGCR, CYP7A1, SREBP2, LDLR, LXRA, and PPARα polyclonal antibodies (Proteintech, Inc., Wuhan, China), and rabbit anti-β-actin (Biosynthesis Biotechnology Co., Ltd., Beijing, China), followed by the secondary antibody, goat anti-rabbit immunoglobulin G/horseradish peroxidase (Goat Anti-rabbit IgG/HRP, Biosynthesis Biotechnology Co., Ltd., Beijing, China). Subsequently, the protein bands were visualized using enhanced chemiluminescence (ECL, Millipore Corp., Billerica, MA, USA) and detected using a chemiluminescence imaging system (Sage Creation, Beijing, China). Lane 1D gel image software (Sage Creation, Beijing, China) was used to quantify the gray scale of protein bands using the value of the β-actin band as a reference.

### Statistical Analysis

All data are expressed as the means ± standard deviation (SD). Differences between groups were compared using a one-way ANOVA test followed by Duncan’s multiple comparison test using SPSS software (SPSS Inc., Chicago, IL, USA).

## Results

### Food Intake, Body Weight Gain, Body Fat Ratio, and Liver Index

**Fig. 3:**
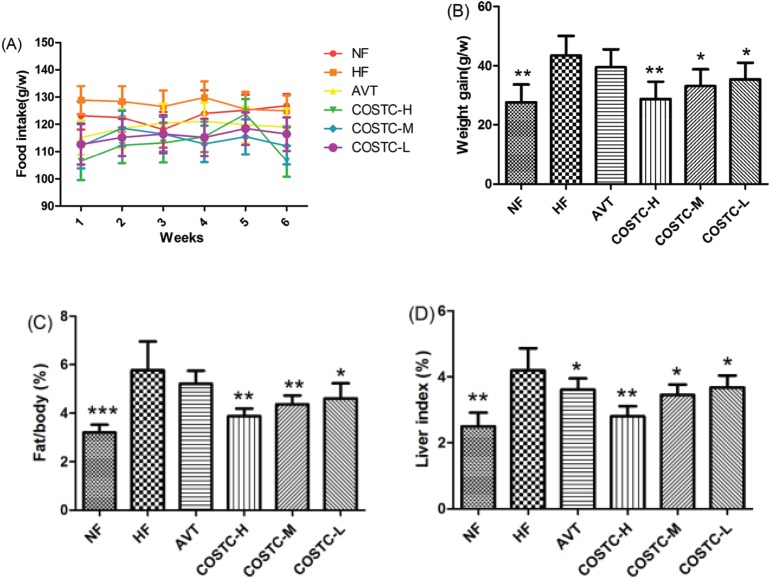
The main index of COSTC. Changes in the food intake (a), body weight gain (b), body fat ratio (c), and liver index (d) after 6 weeks of treatment. The data are presented as the means ± SD (*n* = 10). Note: Compared with HF, * *p* < 0.05; ** *p* < 0.01.

To evaluate the effect of COSTC on cholesterol lowering, the 6-week food intake and body weight gain of rats were analyzed, and the results are shown in Fig. 3. As shown in Fig. 3a, there was no significant difference among the HF, AVT and COSTC groups, indicating that COSTC exerted no influence on the appetite of rats. The result of COSTC on weight gain is revealed in Fig. 3b. In contrast to the HF group, rats administered COSTC-H (*p* < 0.01), COSTC-M, and COSTC-L (*p* < 0.05) showed significantly decreased body weight gains in a dose-dependent manner.

The body fat ratio (Fig. 3c) in the HF group was significantly higher than that for the rats in the NF group (*p* < 0.01), demonstrating that the content of white adipose tissue in HF group was high. COSTC dose-dependently inhibited the high lipid diet-induced elevation of fat pad and body fat ratio, particularly in the COSTC-H and COSTC-M groups (*p* < 0.01), showing effects slightly superior to the AVT group.

The effects of COSTC on liver fat deposition were detected, and the liver indices (liver mass × 100/body mass) are revealed in Fig. 3d. The liver index of the NF group was markedly lower than that of the HF group (*p* < 0.01), indicating that the livers of the HF group possess a high fat content. The liver index was decreased in a dose-dependent manner after administering different doses of COSTC, and the difference of COSTC-H was significant (*p* < 0.01) compared to the HF group. These results demonstrate that COSTC effectively decreased the body weight gain in high-fat-diet-fed rats via preventing the high-fat diet from enhancing the body fat.

### Effect of COSTC on Serum Lipid Levels in Rats

**Fig. 4:**
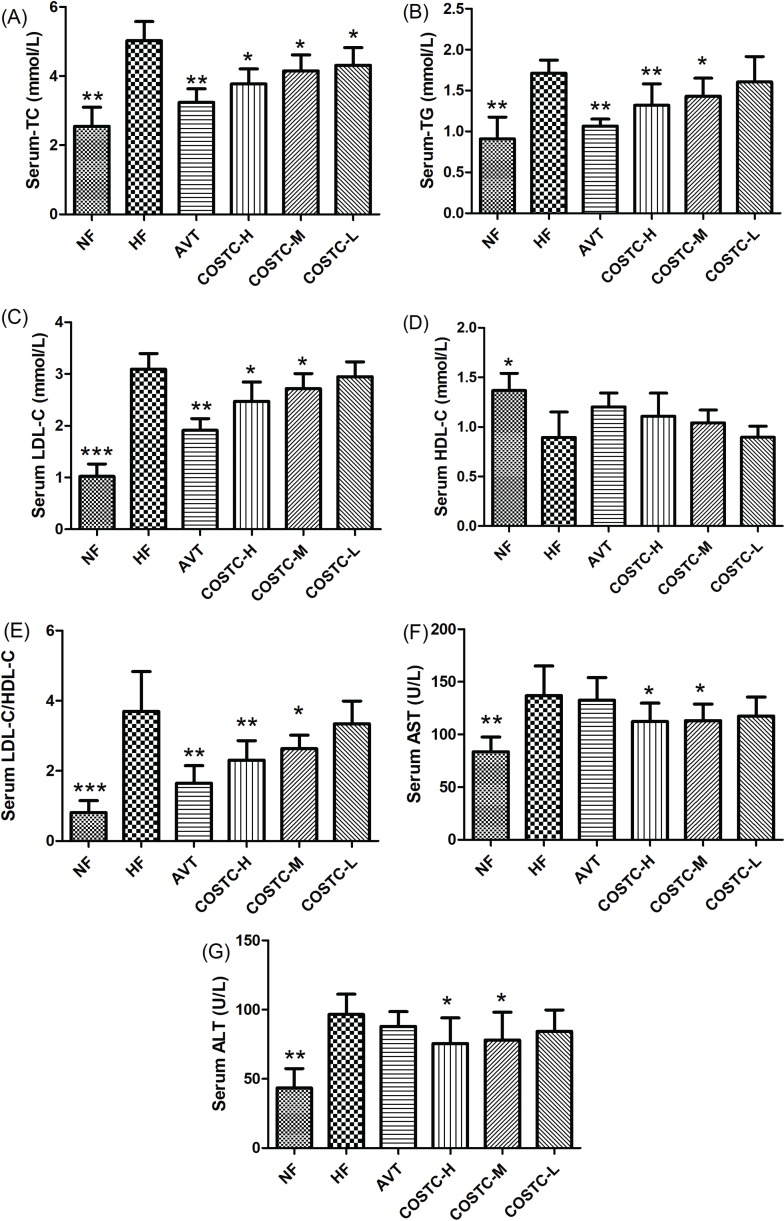
The serum and liver lipid levels in high-fat diet rats. The effect of COSTC on lipid levels in the serum (a-d), AI in the serum (e), and AST activity and ALT activity in the serum (f-g) after 6 weeks of treatment. The data are presented as the means ± SD (*n* = 10). Note: Compared with HF, * *p* < 0.05; ** *p* < 0.01.

The serum lipid levels of high-fat-diet-fed rats were determined using an automatic biochemical analyzer, and the results are displayed in Fig. 4 and Table 1. In this study, the serum and liver TG, TC, and LDL-C levels in the HF group were significantly higher than those in the NF group (*p* < 0.01, Fig. 4a–d), showing that the rat experimental hyperlipidemic model was successful. Compared to the HF group, the serum levels for COSTC-H, COSTC-M, and COSTC-L treatment groups were significantly decreased by 30.50%, 15.85%, and 13.41% for TC, by 26.26%, 19.55%, and 12.85% for TG and by 24.37%, 15.19%, and 7.91% for LDL-C (*p* < 0.05), respectively. However, treatment did not significantly improve the effect on HDL-C.

**Table 1:** The serum, liver, and fetal lipid levels in high-fat diet rats

Groups	NF	HF	AVT	COSTC-H	COSTC-M	COSTC-L
Serum (mmol/L)						
TC	2.55 ± 0.52**	4.92 ± 0.63	3.24 ± 0.37**	3.77 ± 0.41*	4.14 ± 0.44*	4.26 ± 0.48*
TG	0.91 ± 0.27**	1.79 ± 0.15	1.06 ± 0.08**	1.32 ± 0.6**	1.44 ± 0.22*	1.56 ± 0.29*
HDL-C	1.42 ± 0.26*	0.82 ± 0.28	1.06 ± 0.32	1.56 ± 0.35	1.06 ± 0.29	0.96 ± 0.45
LDL-C	1.03 ± 0.24***	3.16 ± 0.25	1.89 ± 0.22*	2.39 ± 0.32*	2.68 ± 0.28*	2.91 ± 0.28*
AI	0.80 ± 0.53***	5.00 ± 0.83	1.56 ± 0.42**	1.65 ± 0.62**	2.06 ± 0.53*	3.44 ± 0.81*
AST (U/L)	83.06 ± 13.86**	138.22 ± 27.98	134.02 ± 21.13	114.20 ± 16.78*	115.10 ± 15.1*	117.88 ± 18.16*
ALT (U/L)	44.88 ± 13.56**	92.88 ± 11.35	85.52 ± 8.76	74.32 ± 18.16*	77.00 ± 20.14*	79.60 ± 15.59*
Fetal (mg/g)						
TC	5.53 ± 1.01**	10.46 ± 1.48	11.67 ± 0.83	15.63 ± 1.76**	14.38 ± 1.56**	12.75 ± 1.80*
TG	5.40 ± 1.42**	12.58 ± 0.96	13.68 ± 1.11	16.16 ± 2.04*	15.48 ± 2.02*	14.63 ± 2.74*
TBA	1.83 ± 0.53**	3.20 ± 0.91	2.90 ± 0.67	5.55 ± 1.53**	5.13 ± 1.51*	4.68 ± 01.43*

TG, triglyceride; HDL-C, high-density lipoprotein cholesterol; LDL-C, low-density lipoprotein cholesterol; NF, control group; HF, feeding group, TC, total cholesterol; TBA, total bile acids; ATV, atorvastatin.

Data are expressed as means ± SD (*n* = 10 per group). Note: Compared with HF;

* *p* < 0.05,

** *p* < 0.01, and

*** *p* < 0.001.

The atherogenic index (AI), an indicator of coronary heart disease risk (28), was significantly reduced in the serum of rats fed COSTC-H (67.00%), COSTC-M (58.80%), and COSTC-L(31.20%) than in that of rats fed HF (*p* < 0.05, Fig. 4E). In addition, the treatment group exhibited a markedly reduced AI value than the HF group in a dose-dependent manner (*p* < 0.05), showing that although COSTC did not significantly ameliorate the effect on LDL-C and HDL-C, it did significantly ameliorate their proportion.

Liver injury, or hepatotoxicity, is the main relative factor of hyperlipidemia (29). Additionally, the increased serum activities of AST and ALT, which were specific toxicological indices for liver function text, are observed in the case of liver injury (30). This study revealed that AST and ALT activities in serum were significantly lower in the COSTC group at a dose of 600 and 300 mg/kg/d than in rats in the HF group (*p* < 0.05, Fig. 4f–g), suggesting that COSTC has no toxicity to the liver function and minimizes the damage resulting from high-fat diet. Thus, that COSTC administration had liver protective activity.

### Effect of COSTC on Fecal TC, TG, and TBA

The positive charge in the structure of chitosan and COS can vigorously absorb fat, fatty acids (FA), and bile acids (BA), containing a negative charge, which contributes to lowering serum TC and TG (31). Nonetheless, several studies have reported that the mechanism through which COSTC reduces lipid levels is related to charge and the regulation of liver lipid metabolism (32). In order to remove excess cholesterol, it must be transported from the peripheral tissue to the liver and intestine, and finally through the feces to the form of BA discharge. Traditionally, the metabolic pathway is thought to be the reverse of the cholesterol transporter or centripetal cholesterol pathway. To determine the effect of the three compounds on the reverse cholesterol transport process, we investigated changes in fecal lipid and BA levels after COSTC administration.

**Fig. 5:**
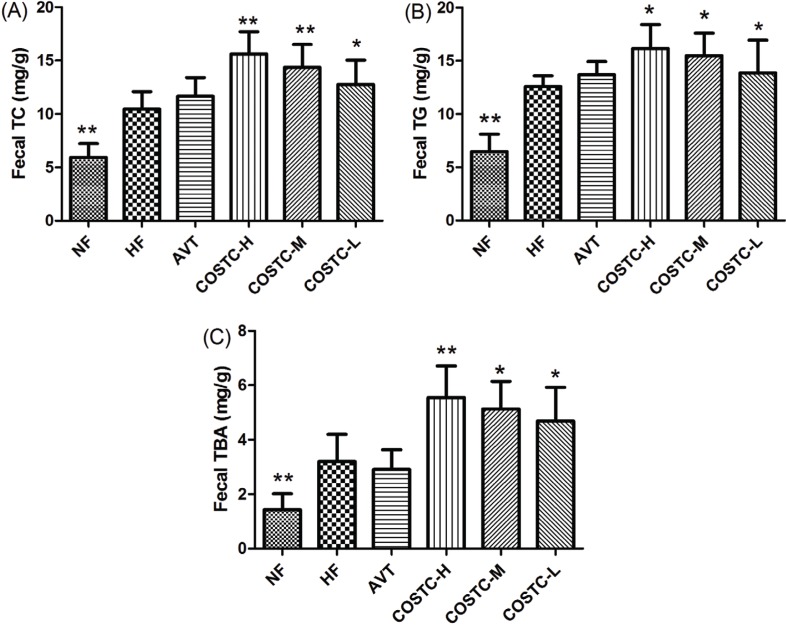
The fecal levels TC, TG, and TBA fetal in high-fat diet rats. The effect of COSTC on fecal levels of TC (a), TG (b), and TBA (c) after 6 weeks of treatment. The data are presented as the means ± SD (*n* = 10). Note: Compared with HF, * *p* < 0.05; ** *p* < 0.01.

The fecal TC, TG, and TBA levels of tested rats are shown in Fig. 5 and Table 1. TC and TG concentrations in rats fed different doses of COSTC increased in a dose- dependently manner compared with that in the HF groups. The results indicated that COSTC effectually reduces the lipid concentration by promoting the excretion of cholesterol via feces.

### Histological Analysis of Different Tissues

**Fig. 6:**
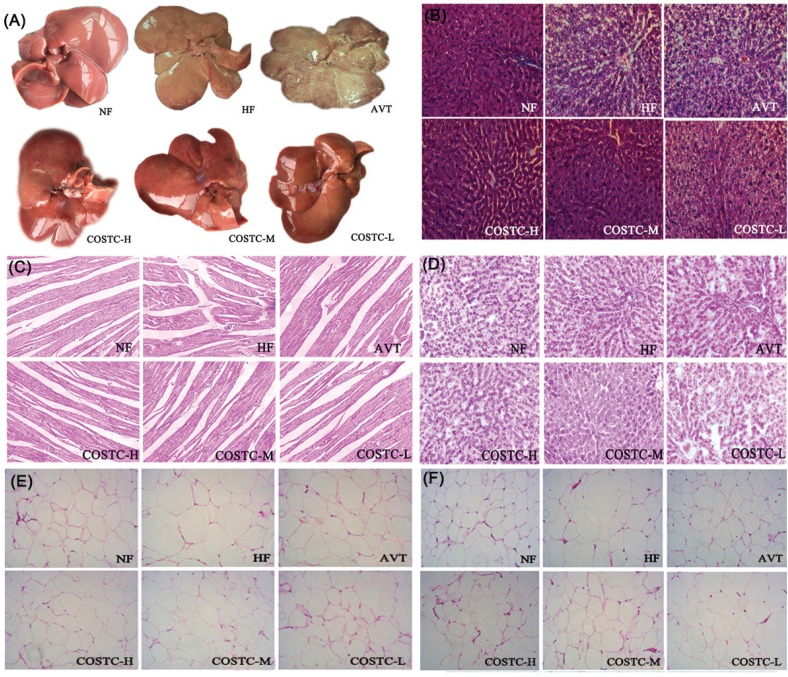
The whole liver (a), slices of liver (b), heart (c), kidney (d), perirenal adipose tissues (e), and subcutaneous adipose tissues (f) from different groups of rats (200×) after 6 weeks of treatment. Tissue sections were stained with hematoxylin and eosin (H&E).

The whole liver and the slices of liver sections are displayed in Fig. 6a–b, respectively. The livers in the NF group were bright red in color, smooth in the tunica of tissues, and characterized by sharp edges, supple texture, and small volume. However, the rats’ livers in the HF group were intumescent and slightly soft; they became pale, the edges were hypertrophic, and the white fat granules in the surface of the liver were readily observed, suggesting that severe fatty-liver-like illness was developed via consuming high-fat diets in rats. The groups administered COSTC improved the steatosis in hepatocytes for which the color was between bright red and dull pale, and the white fat granules were reduced. For further observation, the morphology of liver sections in different groups showed no histological abnormalities in the hepatocytes of the NF group with fewer fat droplets, whereas the hepatocytes in the HF group possessed serious fat vacuoles and partly infiltrated the inflammatory cells, further indicating that the rats developed a high degree of steatosis induced by high-fat diet. Different doses of COSTC groups markedly decreased fat vacuoles in hepatocytes with varying degrees, and the infiltration of inflammation was alleviated, particularly the effects observed in the COSTC-H group, whose cell morphology was similar to that in the NF group, which exerted the hepatoprotective effect via efficiently relieving the fatty liver.

The slices of the heart and kidney pathology tissue were displayed in Fig. 6c–d, respectively. The heart striated muscles and myocardial cells in slices from rats in the HF group (Fig. 6c) were disarrayed with the infiltration of inflammatory cells. However, the heart slices showed aligned myocardial cells without enlargement, clear stripes and muscle space, and have no cardiac hypertrophy in the NF and COSTC-treated groups. In Fig. 6d, the kidney slices from rats treated with different doses of COSTC showed an intact glomerular structure. Additionally, the renal capsule, mesangial cells and matrix, capillary basement membrane, and Sertoli cells of rats in the COSTC groups showed no pathological changes, similar to the NF group. These results demonstrated that the COSTC in different doses were not injurious and were nontoxic in heart and kidney samples.

The images of white adipose pathology tissues sections, including perirenal and subcutaneous fatty tissues, were exhibited in Fig. 6e–f, respectively. The perirenal and subcutaneous adipocytes in the HF group are distinctly hypertrophic compared to the NF group. The sizes of adipocytes were prominently diminished to different degrees after administering different doses of COSTC, suggesting that COSTC can effectively suppress the growth of white adipose tissues to exert the hyperlipidemia effect.

### Differential Gene Expression

**Fig. 7:**
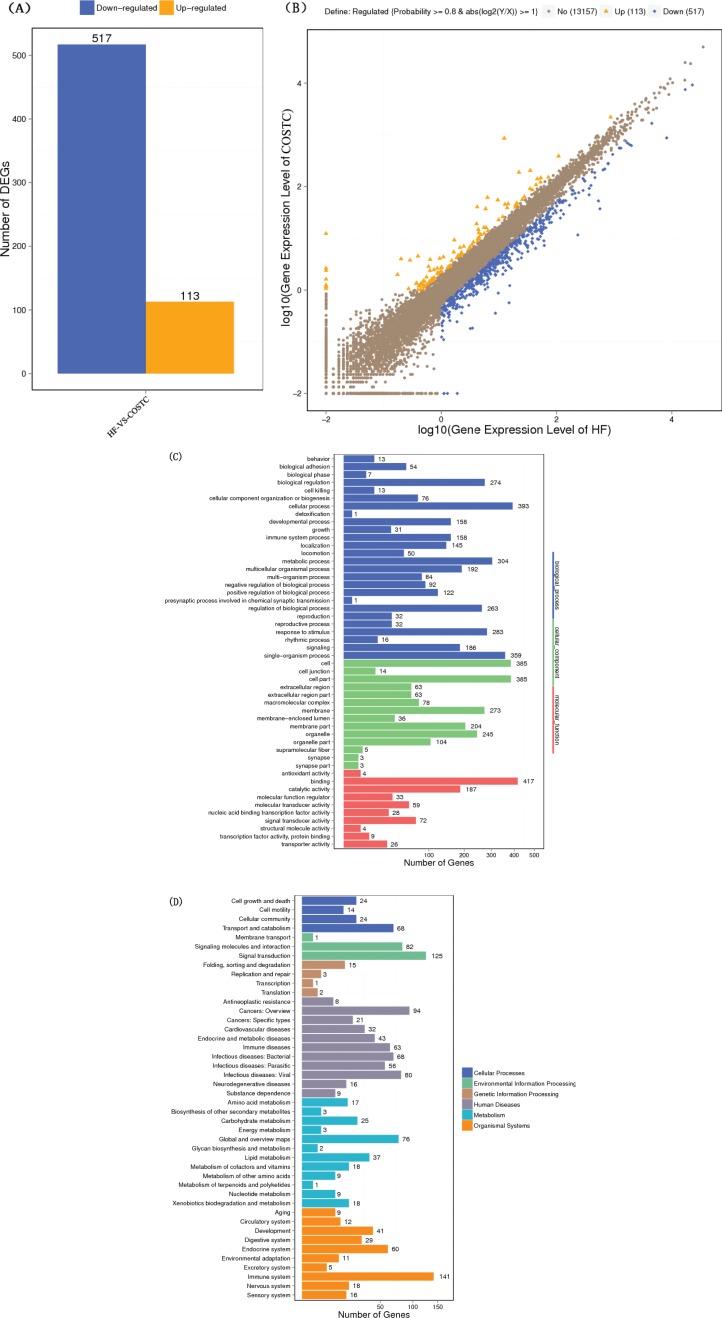
Differential gene expression analysis (HF vs. COSTC).

RNA quality test results showed that the value of 28S/18S was close to 2, indicating that good RNA integrity was consistent with the requirement to construct a sequencing library. The integrity of each RNA sample (RIN) is satisfactory (date not shown). In Fig. 7a–b, the statistical results of differential gene expression analysis were exhibited. Six hundred and thirty genes were differentially expressed in the COSTC group compared to the HF group. Five hundred and seventeen genes were downregulated, while one hundred and thirteen genes were upregulated. The GO enrichment analysis shown in Fig. 7c–d suggested that differentially expressed genes in the COSTC group compared to the HF group were primarily associated with biological regulation and cellular progress, such as organelle membrane, minute bodies, mitochondria and cytoplasm. Among others, the main molecular functions were steroid dehydrogenation activity, oxidoreductase activity, catalytic activity, ion binding, NADH or NADPH as a donor, and purine nucleotide binding. The enriched genes were primarily involved in biological processes, including fatty acid metabolism, lipid metabolism, steroid metabolism, and small molecule metabolites. The genes expressed of large differences (HF vs. COSTC) are shown in Table 2. Based on data analysis, we selected the critical factors involved in fatty acid and lipid metabolism to explore the lipid-lowering activities of COSTC.

**Table 2:** The genes expressed of large differences (HF vs. COSTC)

GeneID	log2 ratio (COSTC/HF)	Upregulation–downregulation (COSTC/HF)	Symbol	GO function or process
25675	0.2244909	Down	HMGCR	GO:0006732;GO:0006694//steroid biosynthetic process;GO:0008203//cholesterol metabolic process; GO:0016114//terpenoid biosynthetic process
691312	10.26795	Up	CYP7A1	GO:0008395//steroid hydroxylase activity; GO:0055092//sterol homeostasis;GO:0050810//regulation of steroid biosynthetic process
100134871	1.8314004	Up	PPARα	GO:0030492//hemoglobin binding; GO:0022892//substrate-specific transporter activity; GO:0016209//antioxidant activity
100314100	8.0407463	Up	LXRα	GO:0005126//cytokine receptor binding; GO:0033764//steroid dehydrogenase activity, acting on the CH-OH group of donors, NAD or NADP as acceptor; GO:0008203//cholesterol metabolic process
500564	5.163333	Up	LDLR	GO:0004871//signal transducer activity; GO:0060090//binding, bridging;GO:0070325//lipoprotein particle receptor binding;GO:0045309//protein phosphorylated amino acid binding
79451	0.176667	Down	SREBP2	GO:0015248//sterol transporter activity; GO:0046983//protein dimerization activity; GO:0055092//sterol homeostasis;GO:0050810//regulaticholesterol synthesis process

HMGCR, 3-Hydroxy-3-Methylglutaryl-Coenzyme A Reductase; HMGCR: 3-Hydroxy-3-Methylglutaryl-Coenzyme A Reductase; PPARα, Peroxisome Proliferation Activated Receptor-α; LXRA, Liver X Receptor Alpha; LDLR, low-density lipoprotein receptor; SREBP-2, Sterol-Responsive Element Binding Protein-2.

### COSTC Inhibits Hepatic Cholesterol Synthesis

**Fig. 8:**
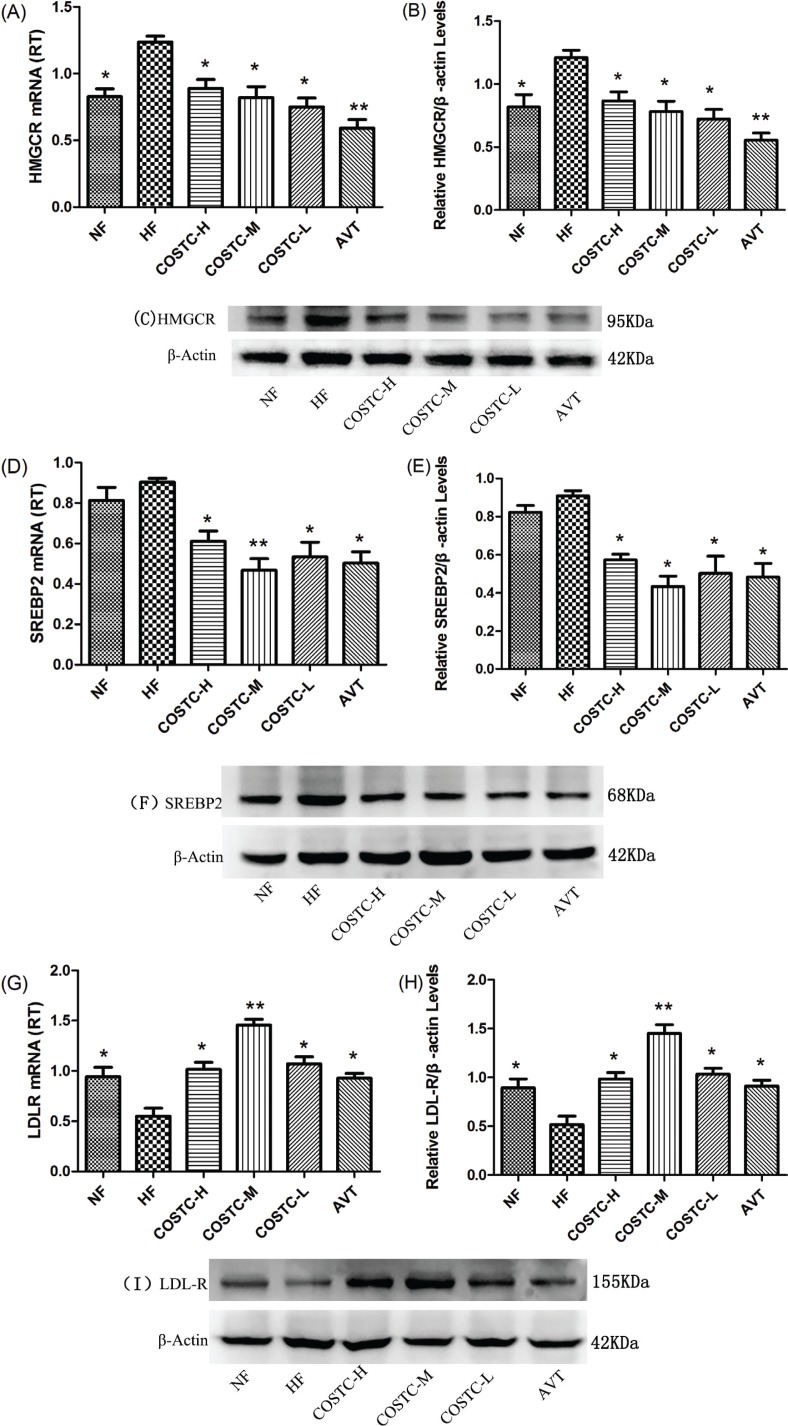
Effect of COSTC on the mRNA and protein expression of hepatic HMGCR genes (a–c); effect of COSTC on the mRNA and protein expression of hepatic SREBP2 genes (d–f); and effect of COSTC on the mRNA and protein expression of hepatic LDLR genes (g–i). The data are the presented as the means ± SD (*n* = 3). Note: Compared with HF, * *p* < 0.05; ** *p* < 0.01.

The first few genes showing differential expression in fatty acid and lipid metabolism were selected for verification, including HMGCR, SREBP2, and LDLR, which are involved in the regulation of hepatic cholesterol synthesis. Therefore, we next investigated these activities using RT-PCR and western blotting. The experimental results presented in Fig. 8a–g showed that the activity of HMGCR and SREBP2 in livers of hyperlipidemic rats was dramatically lower in hyperlipidemic rats following COSTC administration. In contrast, the activity of LDLR in the livers of hyperlipidemic rats administered COSTC was upregulated compared with the HF group (*p* ≤ 0.05). The AVT group showed a similar regulation effect on HMGCR gene expression. Real-time PCR data showed that the gene expression of these modulators in the liver was the same as that of the liver.

### COSTC Promotes Hepatic Cholesterol Excretion

**Fig. 9:**
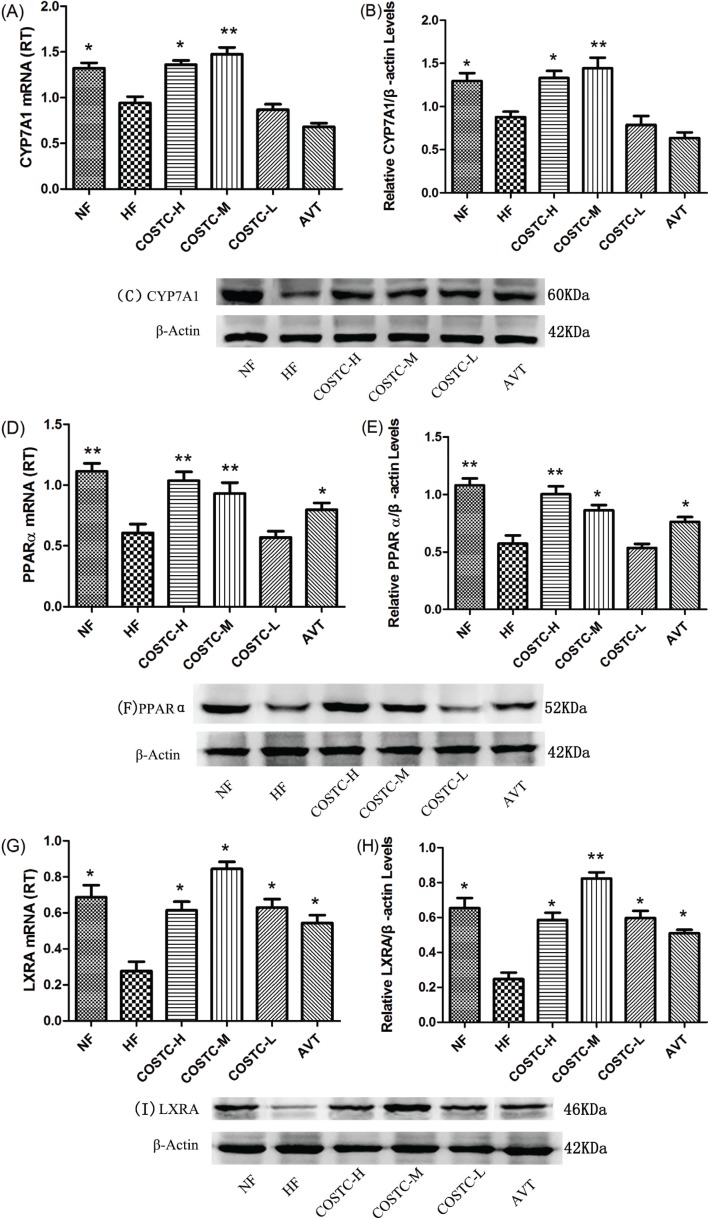
Effect of COSTC on the mRNA and protein expression of hepatic CYP7A1 genes (a–c); effect of COSTC on the mRNA and protein expression of hepatic PPARα genes (d–f); and effect of COSTC on the mRNA and protein expression of hepatic LXRA genes (g–i). The data are presented as the means ± SD (*n* = 3). Note: Compared with HF, * *p* < 0.05; ** *p* < 0.01.

The first few genes showing differential expression in fatty acid and lipid metabolism were selected for verification, including CYP7A1, LXRA, and PPARα, which are involved in the regulation of hepatic cholesterol excretion. Therefore, we next investigated these activities using RT-PCR and western blotting. The experimental results presented in Fig. 9a–g revealed that the activity of CYP7A1, LXRA, and PPARα in the liver of high-fat diet rats was upregulated following the administration of COSTC compared with in the HF group (*p* ≤ 0.05), although COSTC-L did not show significance. The AVT group did not show similar regulatory effect on the gene expression of CYP7A1, as AVT primarily inhibited HMGCR, playing a role in lipid lowering. Real-time PCR data showed that the gene expression of these modulators in the liver was the same as that of the liver.

## Discussion

Previous studies have reported that COS are beneficial in improving plasma lipids and diminishing atherosclerotic risks; as the molecular weight of COS decreases, the biological activity increases (33, 34). In this study, we showed that COSTC administration did not only significantly decrease the serum AST and ALT levels but also reduce the serum TC, TG, and LDLC levels. In addition, we demonstrated that COSTC improved lipid metabolism via upregulating the gene expression and activity of CYP7A1, LXRA, and PPARα, which promote the conversion of cholesterol into bile acid, downregulating the gene expression and activity of enzymes, including HMGCR and SREBP2, and upregulating the gene expression and activity of LDLR to reduce the *de novo* synthesis of cholesterol.

Hyperlipidemia refers to a condition characterized by high levels of cholesterol in the blood (2). These indicators for the clinical diagnostic criteria of hyperlipidemia were examined in this study. An obvious increase in serum TC, TG, and LDL-C levels and a decrease in HDL-C level were observed in hyperlipidemic rats. The regular administration of different doses of COSTC modified the disorders of serum lipid metabolism in 6 weeks, similar to AVT. It is reported that liver injury, or hepatotoxicity, is the main relative factor of hyperlipidemia. Additionally, increased AST and ALT serum activities, which were specific toxicological indices for liver function text, are observed in liver injury. This study showed that serum AST and ALT activities were obviously decreased in the COSTC group at a dose of 600 mg/kg/d than those in the HDF group (*p* < 0.05), suggesting that COSTC has no toxicity to liver function and minimizes the damage induced by high-fat diet. Thus, COSTC administration showed liver protective activity.

Bile acids are synthesized in the liver and secreted into the small intestine where they promote absorption of cholesterol and fat (35). Apart from reabsorbed bile acids, the residue is excreted into the feces (36, 37). In this study, the fecal output of total bile acids was increased in groups administrated with COSTC compared to the HF group, indicating the potential of COSTC to promote the conversion of cholesterol into bile acids in the liver and entrap bile acids in the small intestine, which further increases the excretion of bile acids from feces in addition to the fecal loss of steroids due to reduced enterohepatic recycling. These findings might reflect the potential mechanisms of the cholesterol-lowering effect of COSTC on high-fat-diet-induced hyperlipidemic rats.

The DGE analysis showed that 630 genes were differentially expressed in the COSTC group compared to the HF group, including 517 downregulated genes and 113 upregulated genes. The GO enrichment analysis showed that the enriched genes were primarily involved in biological processes, including bile acids, inflammation, steroid metabolism, and β-oxidation of fatty acids. Based on the analysis of differential expression in fatty acid and lipid metabolism, critical differential gene expressions were selected for verification. CYP7A1, a rate-determining enzyme in the conversion of cholesterol to primary bile acids, catalyzes the initial step in cholesterol catabolism and bile acid synthesis (38). Increased bile acid excretion activates CYP7A1, increasing liver cholesterol into bile acid excretion. This leads to a decrease in free cholesterol levels in the liver, which may in turn stimulate LDLR expression. LXRA is a master regulator maintaining the homeostasis of bile acids (39). PPARa is a nutritional sensor that allows the adaptation of the rates of fatty acid catabolism and lipogenesis (40). SREBP2 is primarily responsible for cholesterol-related gene expression (7). HMGCR is the rate-limiting enzyme of hepatic cholesterol biosynthesis (3). The downregulation of HMGCR and SREBP2 activity will inhibit cholesterol *de novo* synthesis in the liver and thus reduce serum cholesterol levels.

Therefore, in this study, we demonstrated that COSTC improved lipid metabolism via the upregulation of gene expression and activity of CYP7A1, LXRA, and PPARα, which promote the conversion of cholesterol into bile acid, downregulating the gene expression and activity of enzymes including HMG-CoA reductase and SREBP2 and upregulating LDLR to reduce the *de novo* synthesis of cholesterol. Thus, the results of this study indicate that COSTC may be a prospective functional food or supplement for antihyperlipidemic prevention or treatment.

## Conclusions

In summary, this study was the first to provide evidence that COSTC favorably alters lipid metabolism partly through decreased cholesterol biosynthesis and increased cholesterol conversion into bile acids in high-fat-diet-induced hyperlipidemic rats. The potential cholesterol-lowering mechanisms of COSTC could promote the activity of CYP7A1 to increase the cholesterol conversion into bile acids and inhibit the activity of HMGCR to reduce the *de novo* synthesis of cholesterol. Thus, these promising findings indicated that COSTC has potential usefulness as a natural supplement or functional food for preventing and treating hyperlipidemia. Although the in-depth mechanisms need further illumination, these findings will be helpful for designing new therapeutic strategies to prevent the occurrence of hyperlipidemia.
